# The FKBP52 Cochaperone Acts in Synergy with β-Catenin to Potentiate Androgen Receptor Signaling

**DOI:** 10.1371/journal.pone.0134015

**Published:** 2015-07-24

**Authors:** Cheryl Storer Samaniego, Ji Ho Suh, Arundhati Chattopadhyay, Karen Olivares, Naihsuan Guy, Jeffrey C. Sivils, Prasenjit Dey, Fumiaki Yumoto, Robert J. Fletterick, Anders M. Strom, Jan-Åke Gustafsson, Paul Webb, Marc B. Cox

**Affiliations:** 1 Department of Biological Sciences and Border Biomedical Research Center, The University of Texas at El Paso, El Paso, Texas, United States of America; 2 Department of Chemistry and Biochemistry, Kettering University, Flint, Michigan, United States of America; 3 Genomic Medicine, The Methodist Hospital Research Institute, Houston, Texas, United States of America; 4 Department of Biology and Biochemistry and Center for Nuclear Receptors and Cell Signaling, University of Houston, Houston, Texas, United States of America; 5 Department of Biochemistry and Biophysics, The University of California, San Francisco, California, United States of America; Hormel Institute, University of Minnesota, UNITED STATES

## Abstract

FKBP52 and β-catenin have emerged in recent years as attractive targets for prostate cancer treatment. β-catenin interacts directly with the androgen receptor (AR) and has been characterized as a co-activator of AR-mediated transcription. FKBP52 is a positive regulator of AR in cellular and whole animal models and is required for the development of androgen-dependent tissues. We previously characterized an AR inhibitor termed MJC13 that putatively targets the AR BF3 surface to specifically inhibit FKBP52-regulated AR signaling. Predictive modeling suggests that β-catenin interacts with the AR hormone binding domain on a surface that overlaps with BF3. Here we demonstrate that FKBP52 and β-catenin interact directly in vitro and act in concert to promote a synergistic up-regulation of both hormone-independent and -dependent AR signaling. Our data demonstrate that FKBP52 promotes β-catenin interaction with AR and is required for β-catenin co-activation of AR activity in prostate cancer cells. MJC13 effectively blocks β-catenin interaction with the AR LBD and the synergistic up-regulation of AR by FKBP52 and β-catenin. Our data suggest that co-regulation of AR by FKBP52 and β-catenin does not require FKBP52 PPIase catalytic activity, nor FKBP52 binding to Hsp90. However, the FKBP52 proline-rich loop that overhangs the PPIase pocket is critical for synergy.

## Introduction

Androgen receptor (AR)-mediated gene expression contributes to the progression of prostate cancer (PCa), and, for patients with advanced PCa, standard treatments block AR signaling through androgen deprivation or by use of classic AR antagonists. While patients initially respond to treatment, tumors often recur and develop into castration-resistant prostate cancer (CRPC) [1]. As a result, the clinically available therapeutic options including androgen deprivation, classic AR antagonists, and inhibitors of de novo steroidogenesis ultimately fail [2, 3]. Given that AR signaling continues to play a role in CRPC, blocking AR signaling through alternative mechanisms remains a relevant therapeutic strategy. Thus, there is a need for the identification, characterization, and therapeutic targeting of novel molecular mechanisms and regulatory proteins involved in AR activation in PCa.

AR folding and hormone-dependent activation is dependent upon the dynamic, ordered assembly of heteromeric complexes involving multiple chaperone and cochaperone proteins (reviewed in [4]), many of which are potential therapeutic targets for prostate cancer treatment (reviewed in [5]). The final complex in which the receptor is capable of high affinity hormone binding includes the 90-kDa heat shock protein (Hsp90), the p23 cochaperone, and the 52-kDa FK506-binding protein (FKBP52). FKBP52 is one of a family of tetratricopeptide repeat (TPR)-containing proteins that associate with the receptor-Hsp90 complex through direct interaction at the extreme C-terminus of Hsp90. FKBP52 specifically regulates AR, glucocorticoid receptor (GR), and progesterone receptor (PR) activity. Evidence suggests that FKBP52 regulates multiple steps within the receptor signaling pathway, including hormone binding and receptor nuclear translocation [6–10]. In agreement with the biochemical and cellular data, FKBP52 is required for normal male reproductive development and success as *fkbp52*-deficient mice display characteristics of partial androgen insensitivity syndrome including dysgenic prostate [6, 11]. Given the critical role of FKBP52 in AR signaling *in vitro* and *in vivo*, FKBP52 has emerged as an attractive target for the treatment of prostate cancer.

While the FKBP52 peptidyl-prolyl cis/trans isomerase (PPIase) catalytic activity is not required for FKBP52 potentiation of AR activity, the PPIase domain (FK1) is essential. Previous studies demonstrated that the proline-rich loop that overhangs the PPIase catalytic pocket within the FK1 domain is critical for function and likely represents an interaction surface that, at least, transiently contacts the receptor hormone binding domain within the AR-chaperone complex [7, 12]. In addition, mutagenesis studies identified the AR binding function 3 (BF3) surface as the likely site of FKBP52 regulation, and a molecule that is predicted to bind to the BF3 surface termed MJC13 specifically inhibits FKBP52-regulated AR activity [13]. MJC13 prevents hormone-dependent AR dissociation from the chaperone complex, which ultimately inhibits AR translocation to the nucleus, prostate cancer cell proliferation, and growth of prostate tumor xenografts [13–15]. Thus, drugs that target FKBP52 proline-rich loop-AR BF3 interactions represent a novel and promising approach to prostate cancer therapy.

In addition to FKBP52, β-catenin, which is a well-documented regulator of AR-mediated transcription [16–20], has also emerged as an attractive therapeutic target for prostate cancer treatment. Ligand-bound AR competes with transcription factor 4 (TCF4) for regulation by β-catenin [21, 22]. In prostate cancer, free β-catenin regulates AR as a result of activation of wingless (Wnt) signaling pathways [20], and activation of the phosphoinositide 3-kinase (PI3K)/protein kinase B (Akt) pathway is thought to be an upstream regulator of this event. Phosphatase and tensin homolog (PTEN) mutation or loss, and Wnt inhibitory factor 1 (WIF-1) down-regulation are two events that promote Akt activation, which leads to deactivation of glycogen synthase kinase 3β (GSK3β), thereby releasing cytoplasmic β-catenin to participate in nuclear events with AR [23]. Several studies have been conducted to determine residues that are important for direct interaction between β-catenin and AR. β-catenin is capable of binding AR both in the presence or absence of ligand; however, ligand addition increases β-catenin binding to AR in co-immunoprecipitations from LNCaP cell lysates [17]. The armadillo repeats of β-catenin appear to be crucial for its interactions with AR, as found through yeast two-hybrid studies [20] as well as glutathione S-transferase (GST) pull-down assays [19]. In addition, mammalian two-hybrid studies suggested that β-catenin interacts with AR activation function 2 (AF2) [18], a site that is affected allosterically by small molecule binding at the AR BF3 surface [24]. Given that BF3 is the putative binding and/or regulatory site for FKBP52, and both FKBP52 and β-catenin are known positive regulators of AR, we aimed to assess the idea that FKBP52 and β-catenin work in concert to regulate AR activity through the BF3 surface.

## Results

### The predicted β-catenin interaction site overlaps with AR BF3

**Fig 1:**
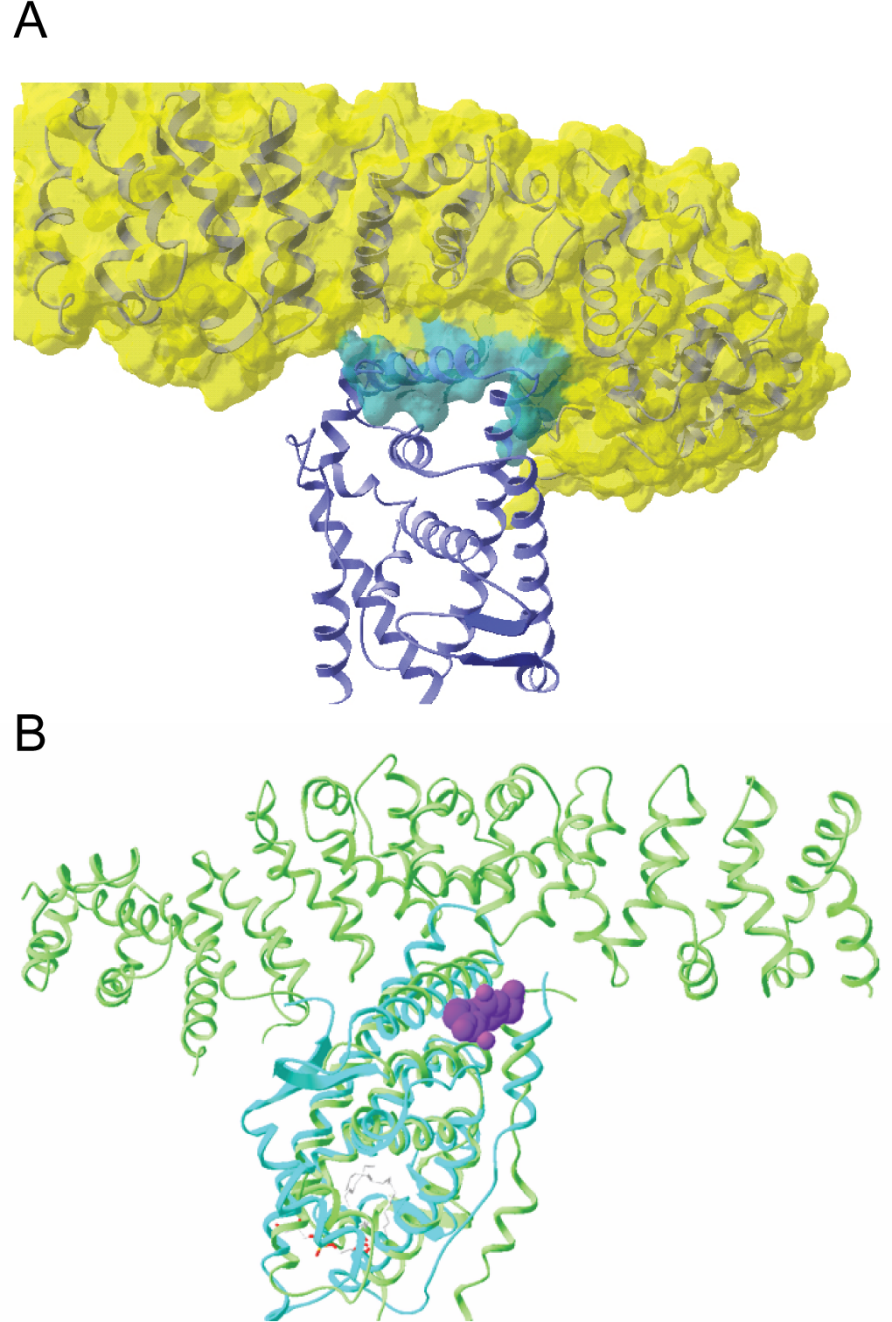
The Predicted β-Catenin Binding Site on AR is Near the Putative FKBP52 Regulatory Surface. (A) Structure of the complex of nuclear receptor LRH-1 with β-catenin, PDB ID 3tx7. β-catenin is shown with a semi-transparent surface in yellow revealing its secondary structure elements as ribbons. LRH-1 is shown with blue ribbons for its secondary structure. The interfacial surface of these two molecules is shown in teal on the β-catenin surface. (B) Flufenamic acid from PDB ID 2PIT is shown in purple spheres as it binds to the androgen receptor shown with its secondary structure as teal colored ribbons. The Cα coordinates of androgen receptor in 2PIT were superimposed with the Cα coordinates of LRH-1 in 3TX7.

Previous studies demonstrated that β-catenin interacts directly with AR to potentiate receptor activity. While the AR ligand binding domain (LBD) and N-terminus were shown to be important for this functional interaction in yeast [20], mammalian two-hybrid studies suggested that β-catenin binds to the AR AF2 to modulate the effects of the N-terminus on ligand-dependent activity [18]. The recently solved co-crystal structure of the liver receptor homolog-1 (LRH-1) hinge-LBD bound to β-catenin demonstrated that LRH-1 and AR bind to a similar surface on β-catenin [25]. Given the high degree of structural similarity in the LBD among members of the nuclear receptor superfamily, it is also likely that LRH-1 and AR share a similar β-catenin interaction surface. Fig 1A illustrates the three-dimensional structure of the complex of LRH-1 LBD bound to β-catenin that was used for modeling the structure of AR LBD with β-catenin. In Fig 1B the crystal structure of the AR LBD is overlaid onto the LRH-1 LBD by superposition of the common Cα atoms. Super positions of atomic coordinates and molecular drawings were done using Swiss-Pdb Viewer [26]. This model predicts that β-catenin interacts with an AR surface that is near the characterized BF3 surface. This surface is highlighted by the presence of flufenamic acid, a known BF3 binding molecule. In this predictive model for AR interaction with β-catenin, flufenamic acid is within 8 Å of β-catenin. Based on this model, we predict that β-catenin interacts with an AR surface that overlaps with the recently characterized BF3 surface [24], which also represents the putative MJC13 binding site on the AR-FKBP52 complex [13].

### FKBP52 directly interacts with β-catenin to promote interaction with AR

**Fig 2:**
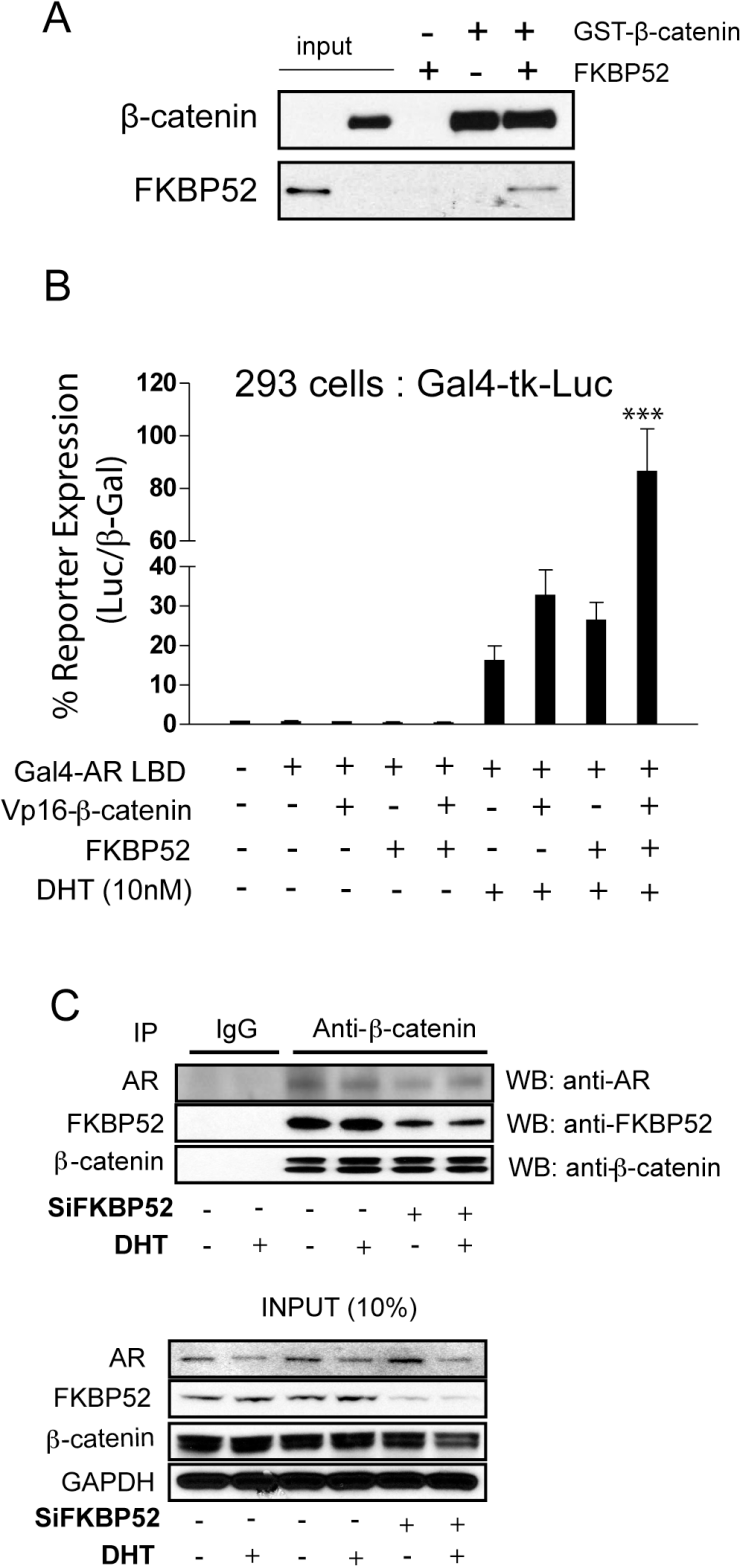
FKBP52 Directly Interacts with β-Catenin to Promote Interaction with AR. (A) *In vitro* GST-pull down assays were performed with purified, recombinant FKBP52 alone, GST-Tagged β-catenin alone, and both recombinant proteins together. Proteins were visualized on Western Blots with primary antibodies specific to human FKBP52 and β-catenin. (B) A mammalian two-hybrid assay assessing the DHT-dependent activity of a Gal4-mediated luciferase reporter in the presence or absence of a Gal4-AR LBD fusion, Vp16-β-catenin and/or FKBP52 demonstrating that FKBP52 potentiates VP-16-β-catenin/AR interaction in 293 cells. Asterisks (***) denote that hormone-dependent reporter expression in the presence of FKBP52, Vp16-β-catenin, and Gal4-AR LBD was significantly enhanced (p values ranging from < 0.01 to < 0.001) as compared to all other conditions. Hormone-dependent reporter expression in the presence of Gal4-AR LBD with FKBP52 or β-catenin alone did not significantly differ (p > 0.05) from the control with Gal4-AR LBD alone. C. A co-immunoprecipitation to detect β-catenin interaction with FKBP52 and AR with and without DHT and FKBP52 siRNA in LNCaP cell lysates. β-catenin was immunoprecipitated and blots probed for AR, FKBP52 or β-catenin. Inputs are shown at bottom. Note that FKBP52 knockdown results in reduced AR/β-catenin interaction despite similar levels of input.

Given that FKBP52 and β-catenin are predicted to co-regulate AR activity through overlapping surfaces we not only assessed the ability of FKBP52 to interact directly with β-catenin *in vitro* in the absence of other proteins (Fig 2A), but also the ability of FKBP52 to influence β-catenin interaction with AR (Fig 2B and 2C). Recombinant human FKBP52 co-precipitated with recombinant GST-tagged β-catenin in a cell-free system, but failed to precipitate on glutathione resin in the absence of GST-tagged β-catenin indicating a direct interaction between FKBP52 and β-catenin in the absence of other proteins (Fig 2A). In addition, the overexpression of FKBP52 in 293T cells synergistically enhanced (4.5-fold) Vp16-β-catenin interaction with Gal4-AR LBD in a mammalian two-hybrid assay as measured by hormone-dependent Gal4-mediated luciferase reporter gene expression, whereas overexpression of Vp16-β-catenin or FKBP52 alone did not significantly enhance hormone-dependent Gal4-responsive reporter expression in the presence of Gal4-AR LBD (Fig 2B). Given that this assay can only assess interactions in the presence of hormone-activated AR LBD, no conclusions regarding the ligand-dependence of the interactions can be gleaned from these data. To further validate these observations, we co-immunoprecipitated β-catenin with AR and FKBP52 from LNCaP prostate cancer cell lysates in the presence or absence of a transiently transfected siRNA targeting FKBP52. While the presence or absence of hormone had no effect on the ability of FKBP52, β-catenin and AR to form a complex, the knockdown of FKBP52 protein levels significantly abrogated β-catenin interaction with AR (Fig 2C). Thus, FKBP52 interacts directly with β-catenin in the absence of other factors and promotes β-catenin interaction with AR independent of ligand.

### FKBP52 is required for β-catenin potentiation of AR activity

**Fig 3:**
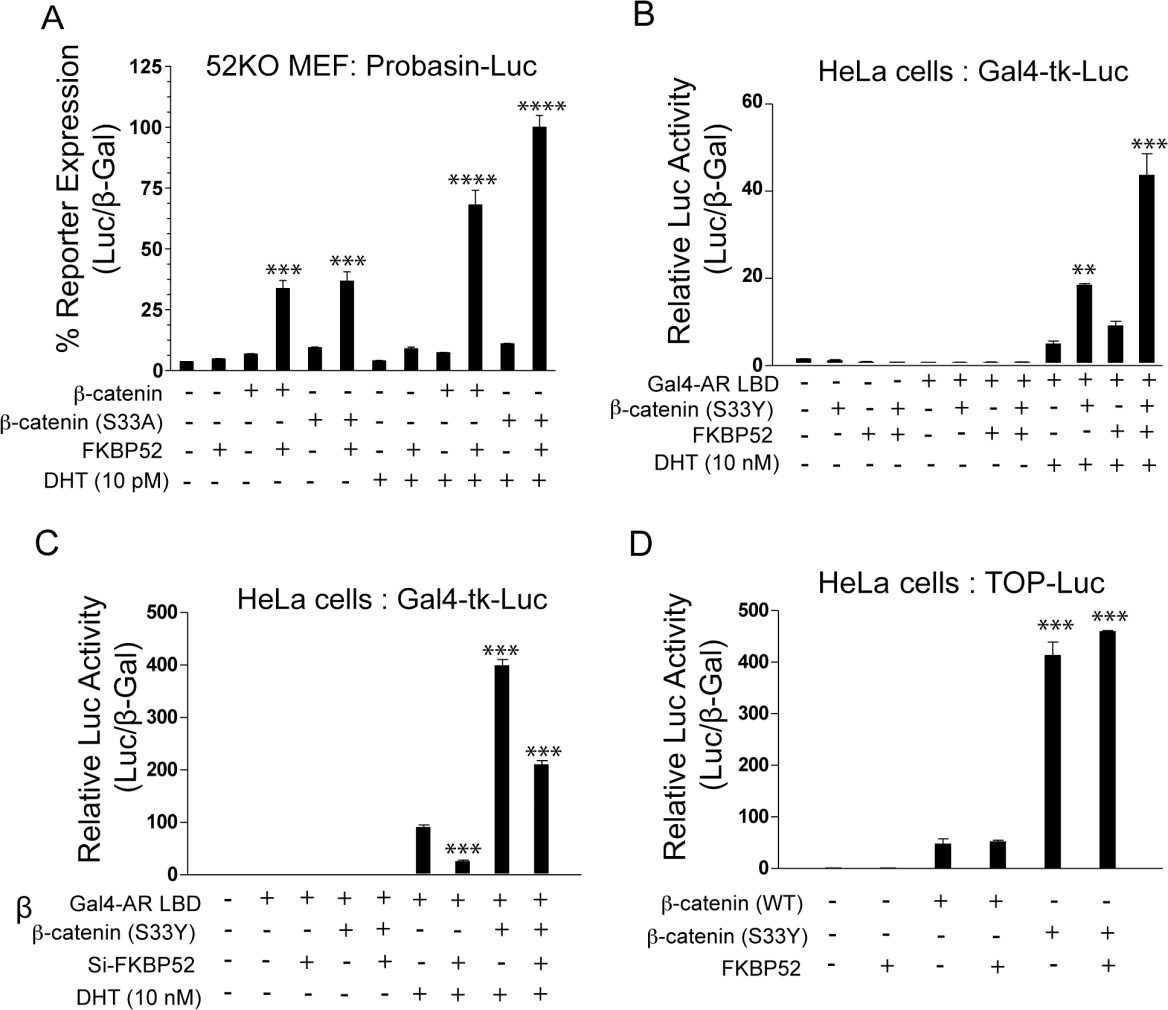
FKBP52 is Specifically Required for β-Catenin Potentiation of AR Activity. (A) AR-mediated luciferase assay in 52KO MEFs in the presence of the indicated transiently transfected expression plasmids with (Black bars) or without (grey bars) dihydrotestosterone (DHT). The asterisks denote a statistically significant difference (***p < 0.001; ****p < 0.0001) as compared to vector alone for each hormone condition. Hormone-dependent receptor activity in the presence FKBP52 and β-catenin also significantly differed as compared to activity in the presence of FKBP52 and β-catenin (S33A) (p < 0.001). The activity in the presence of FKBP52 and wild type or mutant β-catenin was also significantly higher in the presence of hormone than in the absence (p < 0.0001). All other conditions did not significantly differ from the vector alone control, or from each other for each hormone condition. (B) DHT-dependent activity of a Gal4-mediated luciferase reporter in the presence or absence of a Gal4-AR LBD fusion, β-catenin (S33Y) and/or FKBP52 was assessed in HeLa cells. The asterisks denote a statistically significant difference (**p < 0.01; ***p < 0.001) as compared to Gal4-AR LBD alone in the presence of DHT. Hormone-dependent Gal4-AR LBD activity in the presence of both β-catenin (S33Y) and FKBP52 was also significantly (p < 0.001) potentiated as compared to activity in the presence of either β-catenin (S33Y) or FKBP52 alone. (C) The same as in (B), except that transient, siRNA-mediated FKBP52 knockdown was assessed instead of overexpression. The asterisks denote a statistically significant difference (***p < 0.001) as compared to Gal4-AR LBD alone in the presence of DHT. Hormone-dependent Gal4-AR LBD activity in the presence of both β-catenin (S33Y) and Si-FKBP52 was also significantly (p < 0.001) reduced as comparecd to activity in the presence of β-catenin (S33Y) alone. (D) As a control for AR specificity, β-catenin (S33Y) potentiation of TCF4-mediated luciferase activity in HeLa cells was assessed in the presence or absence of FKBP52 overexpression. The asterisks denote a statistically significant (***p < 0.001) potentiation of TCF4-mediated luciferase activity as compared to all other conditions in the absence of β-catenin (S33Y). TCF4-mediated luciferase activity in the presence of β-catenin (S33Y) was not statistically (p > 0.05) different in the presence or absence of FKBP52.

Since the AR BF3 surface is the putative binding and/or regulatory site for FKBP52 [13] and both FKBP52 and β-catenin are known positive regulators of AR, it is possible that FKBP52 and β-Catenin work in concert at this surface. Thus, we aimed to assess the effect of FKBP52 on β-catenin potentiation of AR activity. The *fkbp52*-deficient mouse embryonic fibroblast (52KO MEF) cell line provides a true FKBP52-negative background in which to assess the ability of β-catenin to potentiate AR activity. Thus, we assessed the ability of β-catenin or the degradation-resistant β-catenin (S33A) mutant to potentiate hormone-dependent and hormone-independent AR-mediated luciferase reporter gene expression in the presence or absence of FKBP52 (Fig 3A). While overexpression of β-catenin, or β-catenin (S33A), alone had no significant effect on AR-mediated luciferase expression in this system, the co-expression of FKBP52 with β-catenin, or β-catenin (S33A), resulted in an impressive synergistic up-regulation of both hormone-dependent (17- and 25-fold enhancement respectively as compared to vector alone) and hormone-independent (10-fold enhancement as compared to vector alone) AR activity. Based on these data, we conclude that β-catenin potentiation of AR activity in 52KO MEF cells requires the presence of FKBP52, and that this co-regulation can promote hormone-independent receptor-mediated reporter expression in this system.

To rule out the possibility that the observed effects were unique to the specific reporter system and/or cell line used, we also assessed the effects of FKBP52 overexpression (Fig 3B) and transient siRNA-mediated FKBP52 knockdown (Fig 3C) on β-catenin (S33Y)-mediated potentiation of the Gal4 DNA binding domain (DBD)-AR LBD chimeric protein (Gal4-AR LBD) in the presence of a Gal4-responsive luciferase reporter in HeLa cells. Any effects observed in this system can be attributed to regulation of the AR LBD alone. While the overexpression of either FKBP52 or β-catenin (S33Y) alone in HeLa cells, which endogenously express FKBP52 and β-catenin, resulted in a 1.5 and 4-fold enhancement of Gal4-AR LBD activity respectively, the co-expression of both proteins resulted in a 9-fold enhancement of Gal4-AR LBD activity (Fig 3B). Conversely, siRNA-mediated knockdown of FKBP52 in the presence or absence of β-catenin (S33Y) overexpression in HeLa cells resulted in a 2-fold and 4-fold reduction of Gal4-AR LBD activity respectively as compared with cells overexpressing β-catenin (S33Y), but not treated with siRNA directed against FKBP52 (Fig 3C). Thus, FKBP52 and β-catenin also co-regulate the activity of the Gal4-AR LBD fusion protein in HeLa cells. No enhancement of hormone-independent AR activity was observed in this system, which may reflect the fact that these assays only assess effects on the AR LBD alone only in the presence of hormone. Overexpression of FKBP52 in HeLa cells had no effect on wild type or mutant β-catenin potentiation of the TCF4-responsive TOP-flash reporter plasmid indicating that FKBP52 demonstrates regulatory specificity for β-catenin potentiation of AR activity independent of β-catenin/TCF4-mediated gene transcription (Fig 3D). These data also indicate that the observed effects are not likely due to general effects on transcription, translation, and protein stability.

### FKBP52 and β-catenin co-regulate AR in 22Rv1 prostate cancer cells

**Fig 4:**
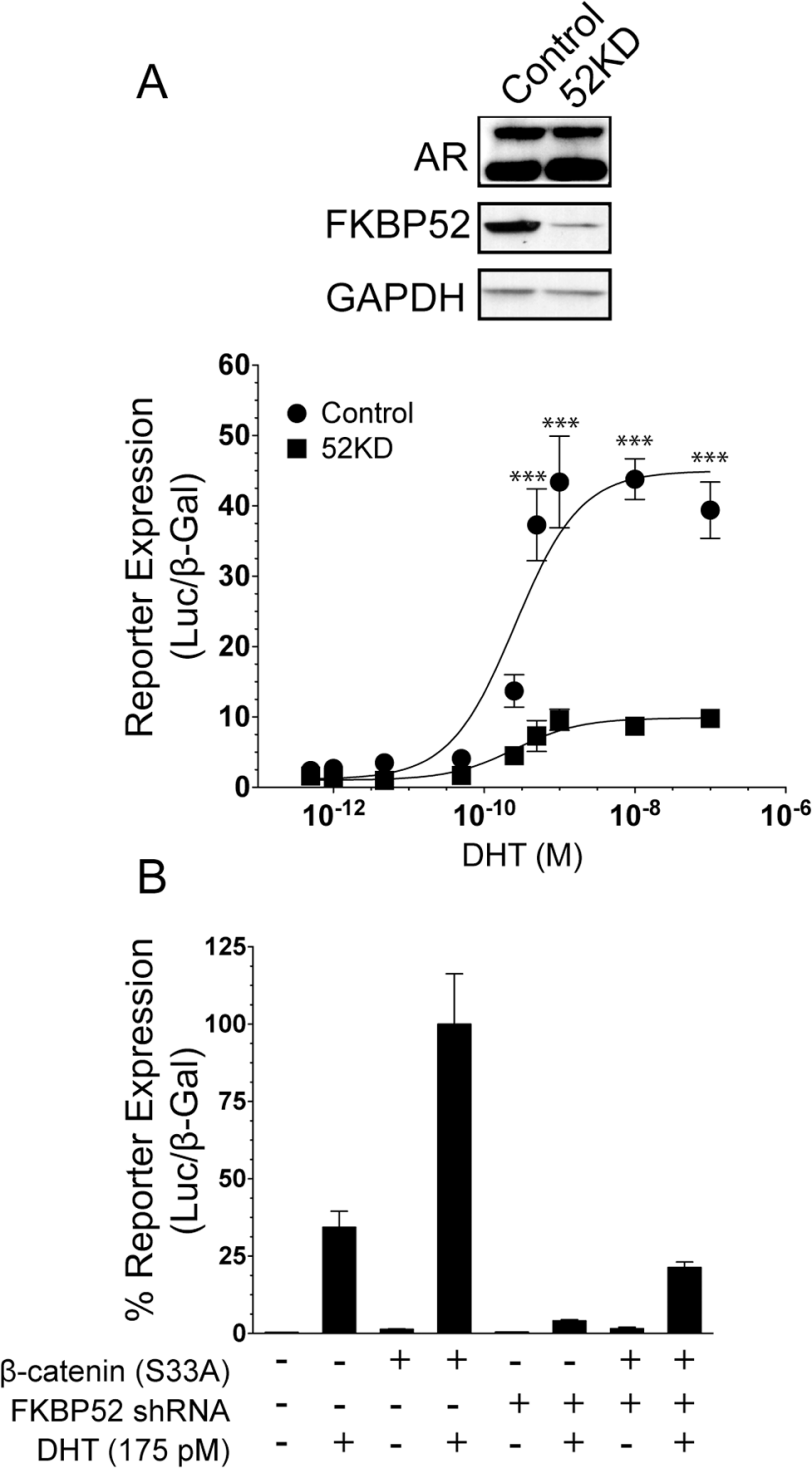
FKBP52 is Required for β-Catenin Potentiation of AR in 22Rv1 Prostate Cancer Cells. (A) AR-mediated probasin-luciferase activity was assessed at a range of hormone concentrations in 22Rv1 prostate cancer cells stably transfected with a 19 base pair shRNA directed against FKBP52 (52KD) or wild type 22Rv1 cells. Significant differences at each hormone concentration are indicated (***p < 0.001). The upper panels show Western blots for AR (both the full length and truncated ARs are shown), FKBP52, and GAPDH as a loading control from 52KD and wild type 22Rv1 cell lysates. (B) A luciferase reporter assay using the AR-inducible probasin-luciferase reporter plasmid in wild type and 52KD 22Rv1 cells with and without overexpression of the indicated proteins in the presence or absence of 175 pM dihydrotestosterone (DHT). Reporter expression in the presence of β-catenin (S33A) and DHT is significantly different from all other conditions (p < 0.001). Wild type cells with empty vector alone in the presence of DHT also significantly differed from all other conditions with p values ranging from < 0.05 to < 0.001. No conditions in the presence of FKBP52 knockdown significantly differed from each other in pairwise comparisons (p>0.05).

The data indicate that FKBP52 and β-catenin co-regulate AR activity in HeLa and 52KO MEF cells. Demonstrating that this co-regulatory mechanism is relevant in a prostate cancer setting required a prostate cancer cell line with stable knockdown of FKBP52 protein in which to assess β-catenin effects on AR activity in the presence or absence of significant levels of FKBP52. Stable knockdown of FKBP52 protein levels was attempted in LNCaP and PC3 prostate cancer cell lines without success. Based on available evidence, the knockdown of FKBP52 is expected to significantly reduce AR signaling leading to a loss of proliferation in AR-dependent prostate cancer cells, which could prevent the selection of clonal populations. While this is true for LNCaP cells, PC3 cells are known to lack AR responsiveness and the reasons for lack of colony formation upon FKBP52 knockdown are unknown. However, we were able to achieve stable, shRNA-mediated FKBP52 knockdown in 22Rv1 prostate cancer cells (Fig 4A, *top panel*), which express both full length AR and a constitutively active, truncated AR protein that supports AR-dependent growth in the absence of full length AR activity. The fact that we were able to achieve knockdown in this cell line suggests that FKBP52 does not regulate activity from the truncated AR protein, which is expected given that FKBP52 is known to act through the receptor LBD [7]. FKBP52 knockdown had no effect on both full length and truncated AR protein levels (Fig 4A, *top panel*), but did significantly reduce hormone-dependent expression of an AR-mediated luciferase reporter gene (Fig 4A, bottom panel). In DHT-treated cells, overexpression of β-catenin (S33A) in wild type 22Rv1 cells, which endogenously express FKBP52, potentiated AR-mediated expression of a luciferase reporter up to 3-fold as compared with the vector control. shRNA-mediated knockdown of FKBP52 led to overall decreased signaling by AR, both in the absence or presence of overexpressed β-catenin. While the overexpression of β-catenin trended towards increased AR activity in the presence of FKBP52 knockdown, this activity was not statistically different from the vector control (Fig 4B). It is interesting to note that the data in Fig 4B were not normalized to hormone-independent basal AR activity. While many, including us, have reported the constitutive AR activity from the truncated AR protein in this cell line, our reporter assays with the probasin promoter consistently lack this basal activity. While we don't have an answer as to why the truncated AR protein does not regulate this reporter construct, this might suggest that the truncated protein displays promoter specificity.

### FKBP52 domain requirements for β-catenin co-regulation of AR

**Fig 5:**
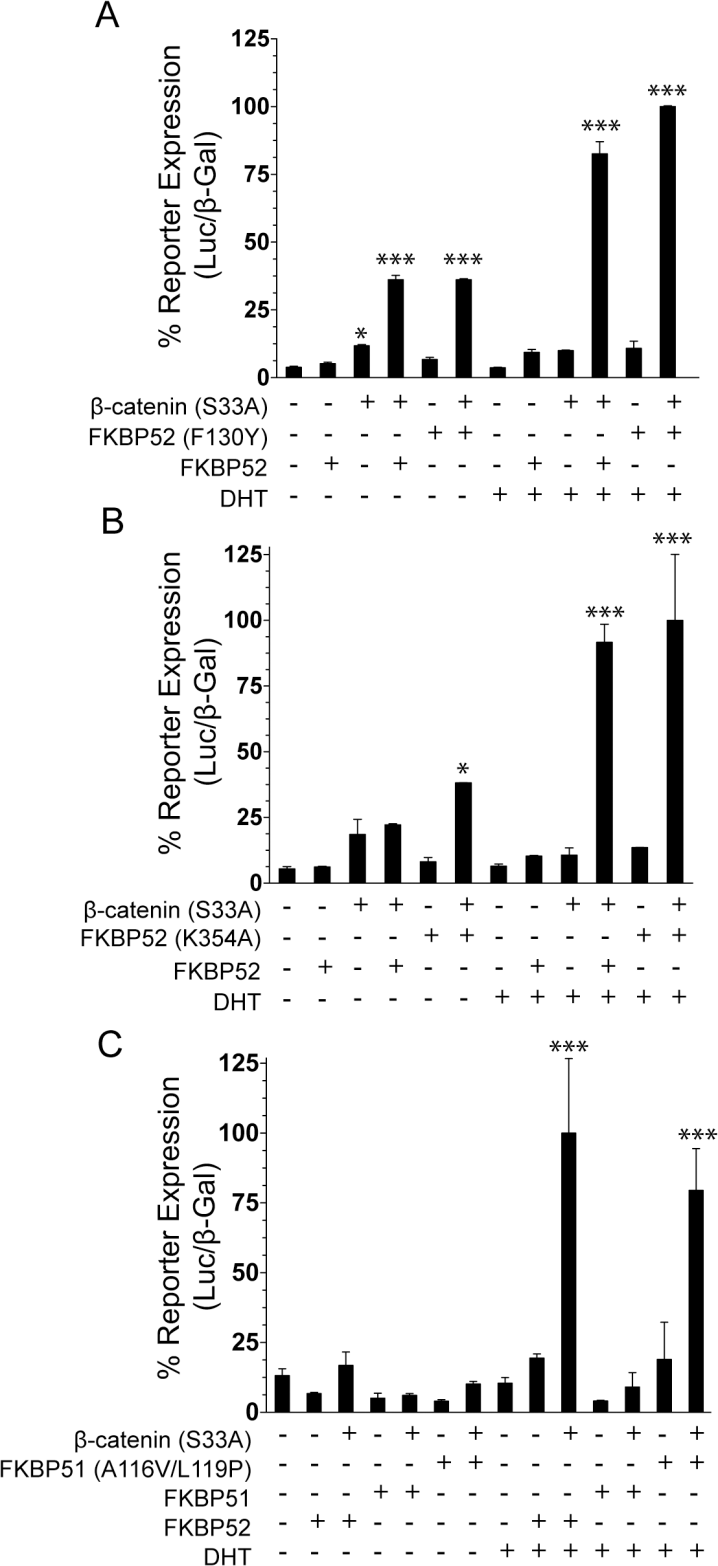
The FKBP52 Domain Requirements for FKBP52/β-Catenin Co-Regulation of AR Activity. (A-C) Wild type AR, the AR-inducible luciferase reporter plasmid, and the constitutively active β-galactosidase reporter plasmid were cotransfected simultaneously with each of the plasmids indicated for the different treatment groups in 52KO MEF cells. Cells were induced with 10 pM DHT or ethanol. Following cell lysis, AR expression was tested through a luciferase assay, followed by normalization to β-galactosidase activity. In all graphs, statistically significant differences as compared to the vector alone control for each hormone condition are denoted by asterisks (*p < 0.05; ***p < 0.001). (A) The assay was performed in the presence or absence of FKBP52, β-catenin (S33A), and the PPIase-deficient FKBP52 mutant FKBP52 (F130Y). The PPIase-deficient FKBP52 mutant retains the ability to synergize with β-catenin (S33A) indicating that PPIase enzymatic activity is not critical for synergy. (B) The assay was performed in the presence or absence of β-catenin (S33A), FKBP52, and the Hsp90 binding-deficient mutant FKBP52 (K354A). FKBP52 binding to Hsp90 is not required for the synergistic upregulation of AR activity by FKBP52 and β-catenin. C. The assay was performed in the presence or absence of β-catenin (S33A), FKBP52, FKBP51, and the FKBP51 (A116V/L119P) mutant. The FKBP51 gain of function mutant exhibits substantial synergism with β-catenin indicating that the FK1 domain and the proline-rich loop are important for synergy.

Given that FKBP52 can potentiate AR activity through interaction with Hsp90, and in the absence of β-catenin in yeast, we aimed to determine if the pre-established domain and functional requirements for FKBP52 potentiation of AR activity alone directly mirror those of FKBP52 co-regulation with β-catenin. While both Hsp90 binding and the FKBP52 proline-rich loop are known to be critical, FKBP52 PPIase catalytic activity is not required [7, 12]. We took advantage of the FKBP52 mutants generated previously, including PPIase-deficient, Hsp90-binding deficient, and proline-rich loop mutants, to characterize the FKBP52 domain requirements for co-regulation with β-catenin using AR-mediated luciferase assays in 52KO MEF cells (Fig 5). The FKBP52 mutation F130Y abrogates PPIase activity without disrupting the conformation of the proline-rich loop, and without inhibiting FKBP52 potentiation of AR activity alone [12]. We assessed FKBP52 (F130Y) for the ability to co-regulate AR activity with β-catenin. The co-expression of FKBP52 (F130Y) with β-catenin (S33A) resulted in the synergistic up-regulation of AR-mediated luciferase reporter expression comparable to that observed with wild type FKBP52 both in the absence and presence of hormone (Fig 5A). Both wild type FKBP52 and FKBP52 (F130Y) overexpression resulted in a 9-fold enhancement of receptor-mediated reporter expression as compared to the vector control in the absence of hormone. Thus, the PPIase-deficient mutant can also support hormone-independent receptor activity. In addition, wild type FKBP52 and FKBP52 (F130Y) overexpression resulted in a 22-fold and 27-fold enhancement of hormone-dependent, receptor-mediated reporter expression respectively. These data are strikingly similar to those reported in Fig 3A. The co-expression of FKBP52 (K354A), which completely lacks Hsp90 binding [7], with β-catenin (S33A) also resulted in the synergistic up-regulation of AR-mediated luciferase reporter expression comparable to that observed with wild type FKBP52 both in the presence and absence of hormone (Fig 5B). In this assay, FKBP52 (K354A) overexpression was able to enhance hormone-independent receptor activity up to 7-fold in addition to enhancing hormone-dependent activity up to 15-fold. It is important to note that in this assay wild type FKBP52 overexpression did enhance hormone-independent receptor activity, but the increase was not statistically significant as compared to vector alone. This reflects the fact that, while a trend for increased hormone-independent activity is present in all assays, this effect is not always consistently significant. In contrast to that observed with FKBP52 alone, these data suggest that both PPIase catalytic activity and FKBP52 binding to Hsp90 are not required for the synergistic up-regulation of both hormone-independent and hormone-dependent AR activity by FKBP52 and β-catenin.

Previous studies demonstrated that two mutations (A116V/L119P) in FKBP51, which does not potentiate AR activity alone, conferred the ability to fully potentiate receptor activity similar to that of FKBP52 [12]. Given that these residues reside within the proline-rich loop, these studies highlighted the importance of the proline-rich loop for potentiation of receptor activity. Interestingly, while FKBP51 lacked the ability to co-regulate with β-catenin (S33A), the co-expression of FKBP51 (A116V/L119P) with β-catenin (S33A) resulted in the synergistic up-regulation of AR-mediated luciferase reporter expression in the presence of hormone (10-fold enhancement as compared to vector alone) (Fig 5C). These data suggest that the FKBP52 proline-rich loop is critical for co-regulation of AR activity with β-catenin. While FKBP51 (A116V/L119P) was capable of synergizing with β-catenin to promote hormone-dependent receptor activity, we observed no statistically significant enhancements of hormone-independent receptor activity, nor a trend for enhancement in the presence of FKBP51 (A116V/L119P). These data may suggest that functional residues and domains on FKBP52 other than the proline-rich loop may contribute to FKBP52/β-catenin potentiation of hormone-independent receptor activity. However, we are cautious in making such a conclusion given that the hormone-independent effects lack consistency.

### MJC13 blocks β-catenin interaction with AR

**Fig 6:**
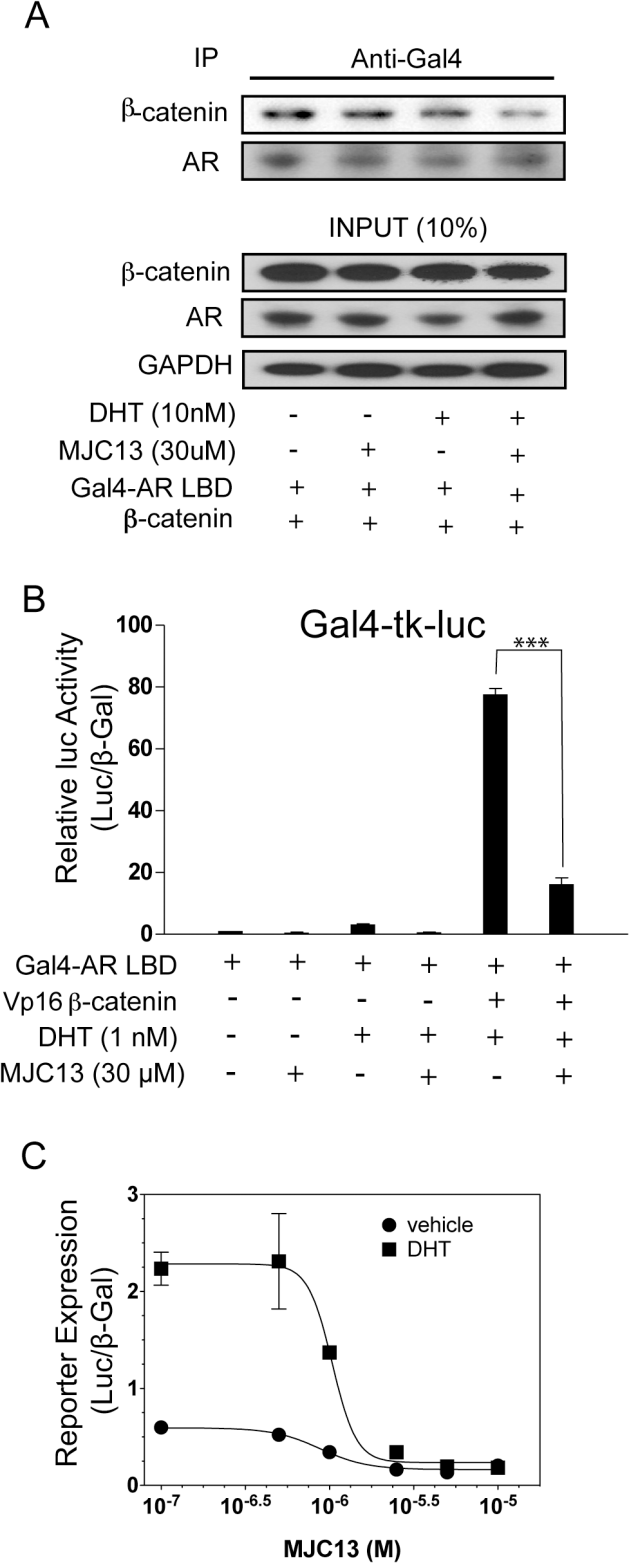
The FKBP52-Specific AR Inhibitor MJC13 Blocks β-Catenin Interaction with AR and Potentiation of AR Activity. (A) A co-IP assay to detect β-catenin interaction with Gal4-AR LBD in 293 cells. Proteins were immunoprecipitated with an anti-Gal4 antibody in lysates from 293 cells treated with or without indicated concentrations of DHT and MJC13. AR LBD was precipitated and blots probed for AR or β-catenin +/- DHT and MJC13. Inputs are shown at bottom. (B) A mammalian two-hybrid assay assessing the DHT-dependent activity of a Gal4-mediated luciferase reporter in the presence of a Gal4-AR LBD fusion with and without Vp16-β-catenin and 30 μM MJC13. MJC13 significantly (***p < 0.001) inhibits hormone-dependent Vp16 β-catenin/Gal4-AR LBD interaction in 293. Both conditions in the presence of Vp16 β-catenin were significantly (p < 0.001) enhanced as compared to hormone-dependent activity in the presence of Gal$-AR LBD alone. (C) 52KO MEFs were co-transfected with FKBP52, β-catenin (S33A), wild type AR, the AR-inducible luciferase reporter plasmid, and the constitutively active β-galactosidase reporter plasmid. After a 1 hour soak with a range of concentrations of MJC13, cells were induced with 10 pM DHT or ethanol for 16 hours. Following cell lysis, AR expression was assessed by luciferase assay. The data represent the average reporter expression (luciferase activity/β-galactosidase activity +/- standard deviation) of four replicates. MJC13 significantly (p < 0.001) inhibited hormone-dependent activity at all concentrations at or above 1 μM. MJC13 significantly inhibited hormone-independent activity at the 2.5 μM (p < 0.05), 5 μM (p < 0.01), and 10 μM (p < 0.05) concentrations.

Our data suggest that FKBP52 promotes β-catenin interaction with AR, and that the β-catenin interaction site overlaps with the AR BF3 surface. Thus, we aimed to determine if the FKBP52-specific AR inhibitor MJC13, which is thought to target the AR BF3 surface [13], could inhibit β-catenin interaction with AR and β-catenin regulation of AR activity (Fig 6). β-catenin was co-immunoprecipitated with Gal4-AR LBD from 293 cell lysates pretreated with and without DHT and MJC13. While the presence of hormone or MJC13 alone had no effect, MJC13 significantly blocked β-catenin interaction with Gal4-AR LBD in the presence of hormone (Fig 6A). In addition, MJC13 significantly abrogated Vp16-β-catenin interaction with Gal4-AR LBD in a mammalian two-hybrid assay as measured by Gal4-mediated luciferase reporter gene expression in 293 cells (Fig 6B). To further validate these findings we assessed the ability of MJC13 to inhibit the synergistic up-regulation of AR-mediated reporter gene expression by FKBP52 and β-catenin in 52KO MEF cells. MJC13 completely inhibited both hormone-independent and hormone-dependent AR activity mediated by FKBP52 and β-catenin at low micro molar concentrations (Fig 6C).

## Discussion

While previous studies suggested that β-catenin binds the AR AF2 [18], a co-crystal structure of β-catenin in complex with the LBD of the orphan nuclear receptor LRH-1 suggests involvement between the fifth armadillo repeat of β-catenin and a surface that overlaps with a region analogous to the AR BF3 surface (Fig 1). Interestingly, the FKBP52 cochaperone is thought to regulate AR through the BF3 surface [13], and our data indicate that FKBP52 directly interacts with β-catenin to promote β-catenin interaction with AR leading to a synergistic up-regulation of AR activity. Given that FKBP52 is ubiquitously expressed in all cell lines and tissues tested to date, all previous functional studies that observed β-catenin co-activation of AR activity were performed in the presence of FKBP52. Our assays in the 52KO MEF cells (Figs 3A and 6) are the first to highlight that the presence of FKBP52 is required for β-catenin to co-activate AR. These data are further validated in the Gal4-tk-Luc model in HeLa cells and in the 22Rv1 prostate cancer cells, where the degree of β-catenin co-activation of AR activity correlates with the level of FKBP52 expression (Figs 3B, 3C and 4).

Our data demonstrate that FKBP52 not only binds directly to β-catenin, but also promotes β-catenin interaction with the AR LBD (Fig 2). Since AR competes with TCF4 for β-catenin binding [21], the presence of FKBP52 may be enough to shift β-catenin to favor AR over TCF4. Indeed, ligand-bound AR decreases the transcriptional activity of TCF4 in prostate cancer, colon cancer [21], and neurons [16]. Because FKBP52 is overexpressed in prostate cancer patients [27], it is feasible that overexpression of FKBP52 could be the cause of increased AR co-activation by β-catenin in prostate cancer. When androgen ablation is utilized as a treatment, β-catenin is thought to shift away from the AR signaling pathway to participate in TCF4 signaling, and this could be a reason for relapse [23]. Additionally, acetylation by p300 at K345 on the sixth armadillo repeat of β-catenin leads to enhancement of β-catenin/TCF4 interactions while causing decreased binding affinity for AR. In prostate cancer, p300 acetylation could either prevent AR binding directly by β-catenin, as suggested by Levy et al. [28], or it could alternatively affect FKBP52 binding to β-catenin, thereby favoring the binding of TCF4 over AR.

Our data suggest that we have discovered a novel mechanism by which FKBP52 and β-catenin regulate the androgen receptor independent of the well-characterized role of FKBP52 within the Hsp90 complex. This is supported by the fact that the receptor specificity for FKBP52 potentiation of receptor function alone does not mirror that for FKBP52 co-regulation of receptor activity with β-catenin. While FKBP52 is known to functionally interact with AR, GR and PR [6–8], β-catenin only interacts strongly with AR, but not GR and PR [20]. GR and PR do interact with Wnt signaling members but not β-catenin directly [29]. The lack of β-catenin interaction with GR and PR is also supported by the fact that we have been unable to observe functional effects on GR and PR activity when β-catenin is overexpressed. In addition to distinct receptor specificity, the co-regulation of AR by FKBP52 and β-catenin also has distinct domain requirements.

The regulation of AR activity by the FKBP52 cochaperone alone has been extensively studied and much has been learned about the FKBP52 domain and residue requirements for function (reviewed in [30]). FKBP52 associates with androgen receptor within the Hsp90 heterocomplex through interaction with the C-terminal EEVD motif in Hsp90. We were unable to detect a direct interaction between FKBP52 and AR LBD *in vitro* in the absence of other proteins. However, evidence suggests that FKBP52 at least contacts the receptor within the context of the Hsp90 multi-chaperone complex. Previous studies in yeast demonstrated that FKBP52 binding to Hsp90 is required for regulation of AR activity [7]. In contrast, our data suggest that FKBP52 co-regulation of AR activity with β-catenin does not require FKBP52 binding to Hsp90 (Fig 5B). While FKBP52 PPIase activity is not required, the proline-rich loop overhanging the PPIase catalytic pocket, which likely represents an interaction surface, is critical for both FKBP52 regulation of AR activity alone [12] and FKBP52 co-regulation with β-catenin (Fig 5A and 5C).

We previously demonstrated that mutations within the AR BF3 surface result in increased dependence on FKBP52 for function, and MJC13, which specifically inhibits FKBP52-regulated AR activity, is predicted to target the AR BF3 surface [13]. Based on these studies the AR BF3 surface, in particular the region containing P723 and F673, has been labeled a putative FKBP52 interaction and/or regulatory surface. Given that FKBP52 binds β-catenin to promote β-catenin binding to the AR LBD (Fig 2), it is possible that FKBP52 regulation through the AR BF3 surface is indirect through interaction with β-catenin. Both FKBP52 and β-catenin may also be part of a larger complex of proteins that acts through the BF3 surface. Indeed, the long isoform of the cochaperone BCL2-associated athanogene (Bag-1L) was recently demonstrated to regulate AR through the BF3 surface and further studies are needed to understand how regulation by Bag-1L is related, if at all, to FKBP52/β-catenin co-regulation [31]. In addition, given that FKBP52 co-regulation with β-catenin is independent of Hsp90 and specific for AR, we propose that FKBP52 potentiation of AR, GR and PR activity within the Hsp90 chaperone complex occurs at a receptor surface that is distinct from BF3. This could also explain why MJC13, which is thought to target the AR BF3 surface, shows specificity for AR as compared to GR. In support of this idea, recent studies suggest that the Helix 1–3 (H1-H3) loop in the GR LBD is important for FKBP regulation and may represent an FKBP52 regulatory site [32]. Thus, FKBP52 likely regulates distinct sites on AR at distinct steps within the AR signaling pathway.

Our data also suggest a role for FKBP52 and β-catenin in promoting hormone-independent AR activity given that FKBP52 synergized with β-catenin to promote AR-mediated expression of a reporter gene in the 52KO MEF cells in the absence of hormone addition (Figs 3A, 5A and 5B), although this effect was not consistently significant between assays (Fig 5B and 5C). This effect was not observed in the Gal4-tk-Luc model in HeLa cells (Fig 3B and 3C), but this system only measures activity from the AR LBD in the presence of hormone and cannot be used to assess hormone-independent effects. We also did not observe effects on hormone-independent AR activity when β-catenin was overexpressed in wild type 22Rv1 prostate cancer cells, a system in which endogenous FKBP52 is present. Thus, no firm conclusions can be drawn from these observations given the inconsistent nature of the data.

Regardless of the manner in which FKBP52 and β-catenin interact to regulate AR activity, our data demonstrate a clear synergistic relationship that is abrogated by treatment with MJC13 (Fig 6). MJC13 is known to specifically inhibit FKBP52-regulated AR activity. However, our data expand the repertoire of MJC13 targets to include both FKBP52- and β-catenin-regulated AR activity. Given that binding at the AR BF3 surface is known to allosterically affect AF2 conformation [24], FKBP52 interaction with β-catenin to regulate AR through the BF3 surface could indirectly affect co-activator binding at AF2. Thus, our data present the possibility that MJC13 targets a variety of factors relevant for the disruption of AR signaling in PCa.

## Materials and Methods

### Plasmids and reagents

Mammalian expression vectors for human FKBP52, β-catenin wild type, β-catenin constitutive active mutant (S33Y), Gal4-AR LBD, Gal4-tk-luc, and TOP-luc were described previously [13, 25]. The FKBP51 and FKBP52 mutants were previously described [12], but the mutations were regenerated directly in the pCI-neo mammalian expression vector using the Quick Change II Site Directed Mutagenesis Kit (Cell Signaling Technologies) according to the manufacturer’s instructions. The degradation-resistant β-catenin S33A mutant was generated directly in the mammalian expression plasmid for β-catenin using the Quick Change II Site Directed Mutagenesis Kit (Cell Signaling Technologies) according to the manufacturer’s instructions. VP16-β-catenin was constructed by inserting an NheI-XbaI digested DNA fragment from pCI-neo-β-catenin into the XbaI digested pACT vector (Promega). Dihydrotestosterone (DHT) and MJC13 were described previously [13, 25].

### Cell culture and transient transfection

22Rv1 prostate cancer cells, 293T fibroblasts, and HeLa cells were obtained commercially (American Type Culture Collection). LNCaP cells were a kind gift from Don Tindall (Mayo Clinic, Rochester, MN) and were originally obtained from the American Type Culture Collection. The *fkbp52*-deficient mouse embryonic fibroblasts (52KO MEF) were previously described [8]. All cells were maintained at 5% CO_2_ in the presence of 10% fetal bovine serum (FBS) unless otherwise indicated. 52KO MEF cells were maintained in Minimal Essential Medium (MEM)/Earl’s Balanced Salt Solution (EBSS) with 2 mM L-glutamine (Thermo Scientific). LNCaP cells were maintained in RPMI 1640 supplemented with L-glutamine. 22Rv1 cells were maintained in RPMI-1640 medium with 25mM HEPES and L-Glutamine (Thermo Scientific), and supplemented with the following reagents, when not provided in medium: 1% Pyruvate (Thermal Scientific), 1% MEM-non essential amino acids (Thermal Scientific), and 1% Pen-Strep (Thermal Scientific). HeLa cells and 293T cells were maintained in Dulbecco's modified Eagle's medium (DMEM). 24 hours prior to transfection all cells were switched to medium containing Charcoal/Dextran Treated Fetal Bovine Serum (FBS) (HyClone). For transfection of 52KO MEF, 22Rv1 and 52KD 22Rv1 cells, cells were plated in 6-well plates and allowed to reach 80% confluence prior to transfection. Cells were transfected with Lipofectamine 2000 (Invitrogen Life Technologies) at a DNA to lipofectamine ratio of 1:3 in MEM-EBSS lacking FBS according to the manufacturer’s instructions. Typically, transfections included a constitutive β-galactosidase reporter plasmid (pCMVβ; Clonetech) as a transfection control, a firefly luciferase reporter plasmid (pT81, American Type Culture Collection) driven by the androgen-dependent probasin promoter, a human AR expression plasmid, and plasmids expressing the indicated FKBP and β-catenin variants, or empty parent vector control. For transient transfection of HeLa, LNCaP, and 293T cells, cells were plated in 24-well plates and transfected with expression plasmids, reporter plasmid, and the control β-galactosidase expression plasmid (pCMV-β) using Fugene HD Transfection reagent (Roche) according to manufacturer’s instructions. Total amounts of expression vector plasmids were kept constant by the addition of appropriate amounts of empty pCMV vector. The siRNA for FKBP52 (ID no. s48) was purchased from Ambion. HeLa cells and LNCaP cells were transfected with siRNA (100 nM) using Lipofectamine RNAiMAX reagent (Invitrogen Life Technologies) according to manufacturer’s instructions.

### Stable FKBP52 knockdown in 22Rv1 cells

To generate stable FKBP52 knockdown in 22Rv1 prostate cancer cells, an shRNA specific for FKBP52 and scrambled shRNA control [6] were subcloned into the pSilencer 2.1-U6 hygro plasmid (Invitrogen Life Technologies) according to the manufacturer’s recommendations. Cells were transfected with 1 μg/35 mm well using Lipofectamine 2000 (Invitrogen Life Technologies) in Opti-MEM medium (Invitrogen Life Technologies) according to the manufacturer’s protocol. Complete RPMI 1640 medium with 10% FBS medium was added after 6 h of incubation with the transfection reagents. After 48 hours 200 μg/ml hygromycin was added and after two weeks single clones were picked and expanded. The hygromycin concentration for selection was determined by titration prior to the beginning of the experiment. The absence of FKBP52 protein was assessed by Western immunoblot as described below.

### Luciferase reporter assays

For all reporter assays systems, the single hormone concentrations used were determined from hormone dose-response curves. For assessing FKBP52 and/or β-catenin regulation we chose a dose near the bottom of the curve to maximize the observed AR potentiation. Twenty-four hours after transfection, medium was replaced with medium containing the indicated concentrations of dihydrotestosterone (DHT). After approximately 16 hours of incubation with hormone, cells in each well were lysed using 100μL mammalian protein extraction reagent (M-PER) (Pierce) supplemented with Complete ethylenediaminetetraacetic acid (EDTA)-free Mini Protease Inhibitor (Roche) and clarified in a microcentrifuge. Approximately 100 μl of lysate from each experimental condition was used for the luciferase assay and the remainder was used for Western Immunoblots as described below. AR-mediated luciferase expression was quantified by mixing 40 μL of cell lysate with 100 μL of luciferase assay reagent (Promega) in a 96-well plate. β-galactosidase expression was quantified by adding 20 μL cell lysate with 100 μL of Tropix Gal-Screen (Applied Biosystems). The 96-well plates were incubated at room temperature (incubation time was 5 minutes for the luciferase assay and 2 hours for the β-galactosidase assay), followed by quantification of luminescence in a microplate luminometer (Luminoskan Ascent, Thermo Labsystems). Luminescence was measured in Relative Light Units (RLU) and normalized to β-galactosidase activity (luciferase RLU/ β-galactosidase) to control for transfection efficiency. Finally all data were normalized to a percentage of the maximum luciferase activity. All figures are representative of at least three independent experiments with similar results. Where indicated, statistically significant differences were determined by one-way analysis of variance followed by pair-wise comparisons using Bonferroni multiple comparisons tests with a 95% confidence interval. For assays with the Gal4-tk-Luc reporter in HeLa cells, cells maintained in 10% charcoal-stripped serum were treated with or without 10 nM dihydrotestosterone (DHT) and 30μM MJC13 for 24 h following transfection. Luciferase and β-galactosidase activities were assayed as previously described [25]. The levels of luciferase activity were normalized to β-galactosidase expression.

### Mammalian two-hybrid assay

Mammalian two-hybrid assay was performed with 293T cells. Plasmids for Gal4-DBD fused to the AR LBD and plasmids for VP16-AD fused to the full-length β-catenin with Gal4-tk-luc reporter were cotransfected into 293T cells. Cotransfected cells maintained in 10% charcoal-stripped serum were treated with and without 10 nM DHT and 30**μ**M MJC13 for 24 h following transfection. Luciferase and β-galactosidase activities were assayed as previously described [25]. The levels of luciferase activity were normalized to β-galactosidase expression.

### Co-immunoprecipitations

Co-immunoprecipitations were performed from extracts of LNCaP cells that were transfected with FKBP52 siRNA or 293T cells that were co-transfected with Gal4-AR LBD and β-catenin expression plasmids. Transfected or co-transfected cells maintained in 10% Charcoal-stripped serum were treated with and without 10 nM DHT and 30**μ**M MJC13 following transfection, and harvested in RIPA cell lysis buffer (50 mM Tris-HCl, pH 7.5, 150 mM NaCl, 2.5 mM EGTA, 1% NP-40, Protease inhibitor cocktail (Roche)). Whole-cell lysate was incubated with 2 **μ**g of anti-β-catenin or anti-Gal4 antibody (Santa Cruz Biotechnology) for 4 hours at 4ºC, and was further incubated for another 12 hours after the addition of 30 **μ**l of protein A/G agarose bead slurry (Santa Cruz Biotechnology). Agarose beads were washed three times with RIPA buffer at 4ºC, and bound proteins were separated by SDS-PAGE. Proteins on the gels were transferred to a PVDF membrane, subjected to Western blot analysis with anti-AR (Santa Cruz Biotechnology), anti-FKBP52 (Cell Signaling Technologies), anti-β-catenin (Santa Cruz Biotechnology), and anti-GAPDH (Cell Signaling Technologies) antibodies, and then detected with an ECL kit (Amersham Pharmacia).

### GST pull-down assay

For the GST pull-down assays 20 **μ**L of Glutathione-Sepharose 4B (GE Healthcare, Sweden) suspended resin per assay condition was added to microcentrifuge tubes, centrifuged at 4°C, and the supernatant was removed. Resin beds were washed in ice-cold Binding Buffer (50mM HEPES pH 7.4, 50mM KCl, 10mM MgCl_2_, 0.01% Tween 20, 1 mM DTT) supplemented with EDTA free, Halt protease inhibitor cocktail (Pierce, Rockford, IL) and resuspended in 400 **μ**L of Binding Buffer. Recombinant GST-Tagged β-catenin (Millipore, Tamecula, CA) or buffer control was preloaded onto the resin by gently mixing by inversion for 1.5 hours at 4°C. All tubes were centrifuged at 4°C and the supernatant was removed. The resin beds were then washed three times with ice-cold Wash Buffer (50mM HEPES pH 7.4, 50mM KCl, 10mM MgCl_2_, 0.08% Tween 20, 1 mM DTT) supplemented with EDTA free, Halt protease inhibitor cocktail (Pierce). Samples were resuspended in 400 **μ**L Binding Buffer and an equimolar amount of recombinant C-terminal 6xhistidine-tagged FKBP52 protein or buffer control was added where indicated. The purification of functional 6xhistidine-tagged FKBP52 was previously described [33]. All samples were gently mixed by inversion for 1.5 hours at 4°C and then clarified. Samples were washed in Wash Buffer three times and 15 **μ**L of beta-mercaptoethanol/4 x sodiumdodecylsulfate solution was added to each sample, including input samples of FKBP52 and β-catenin protein, and all samples were heat denatured at 95°C for 5 minutes. Proteins were separated by electrophoresis, transferred to Immobilon polyvinylidene fluoride (PVDF) membranes (Millipore), and Western immunoblots were performed as detailed below.

### Western immunoblot

Western immunoblots were performed using the prepared lysates from assays as described above. Total cellular protein concentrations were determined by Coomassie Plus (Bradford) Protein Assay (Thermo Fisher Scientific Inc., Rockford, IL). Equivalent amounts of protein were loaded for each sample on 10–20% Criterion gels (BioRad), proteins were separated by electrophoresis, and proteins were transferred to Immobilon PVDF membranes (Millipore) following standard procedures. The following primary antibodies were used to detect proteins of interest: Rabbit polyclonal anti-β-catenin (Millipore), mouse monoclonal anti-FKBP52 (Hi52D, epitope in the FK1 domain) [34], rabbit polyclonal anti-AR (N-20; Santa Cruz Biotechnology), and mouse anti-glyceraldehyde-3-phosphate dehydrogenase (GAPDH) (Biodesign International) as a loading control. The secondary antibodies used included AP-conjugated goat anti-rabbit and anti-mouse IgG secondary antibodies (Southern Biotechnology). The ImmunStar Alkaline Phosphatase Substrate (BioRad) was applied prior to exposing to X-ray films for development and detection of antibodies.

## References

[1] Decker KF, Zheng D, He Y, Bowman T, Edwards JR, Jia L. Persistent androgen receptor-mediated transcription in castration-resistant prostate cancer under androgen-deprived conditions. Nucleic Acids Res. 2012;40(21):10765–79. Epub 2012/09/29. 10.1093/nar/gks888 23019221 PMC3510497

[2] Yamaoka M, Hara T, Kusaka M. Overcoming persistent dependency on androgen signaling after progression to castration-resistant prostate cancer. Clin Cancer Res. 2010;16(17):4319–24. Epub 2010/07/22. 10.1158/1078-0432.CCR-10-0255 . 20647476

[3] Wang J, Shang Z-q, Niu Y-j. Androgen receptor roles in benign and malignant prostate disease. Clinical Oncology and Cancer Research. 2011;8(2):85–91. 10.1007/s11805-011-0564-x

[4] Smith DF, Toft DO. Minireview: the intersection of steroid receptors with molecular chaperones: observations and questions. Mol Endocrinol. 2008;22(10):2229–40. Epub 008 May 1. 10.1210/me.2008-0089 18451092 PMC2582531

[5] Cano LQ, Lavery DN, Bevan CL. Mini-review: Foldosome regulation of androgen receptor action in prostate cancer. Mol Cell Endocrinol. 2013;369(1–2):52–62. Epub 2013/02/12 06:00. 10.1016/j.mce.2013.01.023 23395916

[6] Cheung-Flynn J, Prapapanich V, Cox MB, Riggs DL, Suarez-Quian C, Smith DF. Physiological role for the cochaperone FKBP52 in androgen receptor signaling. Mol Endocrinol. 2005;19(6):1654–66. . 15831525 10.1210/me.2005-0071

[7] Riggs DL, Roberts PJ, Chirillo SC, Cheung-Flynn J, Prapapanich V, Ratajczak T, et al The Hsp90-binding peptidylprolyl isomerase FKBP52 potentiates glucocorticoid signaling in vivo. EMBO J. 2003;22(5):1158–67. . 12606580 10.1093/emboj/cdg108 PMC150341

[8] Tranguch S, Cheung-Flynn J, Daikoku T, Prapapanich V, Cox MB, Xie H, et al Cochaperone immunophilin FKBP52 is critical to uterine receptivity for embryo implantation. Proc Natl Acad Sci U S A. 2005;102(40):14326–31. Epub 2005 Sep 21. 16176985 10.1073/pnas.0505775102 PMC1242310

[9] Erlejman AG, Lagadari M, Harris DC, Cox MB, Galigniana MD. Molecular chaperone activity and biological regulatory actions of the TPR-domain immunophilins FKBP51 and FKBP52. Curr Protein Pept Sci. 2014;15(3):205–15. Epub 2014/04/04. CPPS-EPUB-59873 [pii]. . 24694367 10.2174/1389203715666140331113753

[10] Sivils JC, Storer CL, Galigniana MD, Cox MB. Regulation of steroid hormone receptor function by the 52-kDa FK506-binding protein (FKBP52). Curr Opin Pharmacol. 2011;11:314–319. 10.1016/j.coph.2011.03.010 21511531 PMC3156321

[11] Yong W, Yang Z, Periyasamy S, Chen H, Yucel S, Li W, et al Essential role for Co-chaperone Fkbp52 but not Fkbp51 in androgen receptor-mediated signaling and physiology. J Biol Chem. 2007;282(7):5026–36. Epub 2006 Dec 1. 17142810 10.1074/jbc.M609360200 PMC2577319

[12] Riggs DL, Cox MB, Tardif HL, Hessling M, Buchner J, Smith DF. Noncatalytic role of the FKBP52 peptidyl-prolyl isomerase domain in the regulation of steroid hormone signaling. Mol Cell Biol. 2007;27(24):8658–69. Epub 2007 Oct 15. 17938211 10.1128/MCB.00985-07 PMC2169416

[13] De Leon JT, Iwai A, Feau C, Garcia Y, Balsiger HA, Storer CL, et al Targeting the regulation of androgen receptor signaling by the heat shock protein 90 cochaperone FKBP52 in prostate cancer cells. Proc Natl Acad Sci U S A. 2011;108(29):11878–83. Epub 2011/07/07. 1105160108 [pii] 10.1073/pnas.1105160108 21730179 PMC3141981

[14] Liang S, Bian X, Liang D, Sivils JC, Neckers LM, Cox MB, et al Solution formulation development and efficacy of MJC13 in a preclinical model of castration-resistant prostate cancer. Pharm Dev Technol. 2014:1–6. Epub 2014/11/08. 10.3109/10837450.2014.979946 . 25380396 PMC4428990

[15] Liang S, Bian X, Sivils J, Neckers LM, Cox MB, Xie H. Quantification of a New Anti-Cancer Molecule MJC13 Using a Rapid, Sensitive, and Reliable Liquid Chromatography-Tandem Mass Spectrometry Method. American Journal of Modern Chromatography. 2014;1(1):1–11. 25594071 PMC4292881

[16] Pawlowski JE, Ertel JR, Allen MP, Xu M, Butler C, Wilson EM, et al Liganded androgen receptor interaction with beta-catenin: nuclear co-localization and modulation of transcriptional activity in neuronal cells. J Biol Chem. 2002;277(23):20702–10. . 11916967 10.1074/jbc.M200545200

[17] Truica CI, Byers S, Gelmann EP. Beta-catenin affects androgen receptor transcriptional activity and ligand specificity. Cancer Res. 2000;60(17):4709–13. . 10987273

[18] Song LN, Herrell R, Byers S, Shah S, Wilson EM, Gelmann EP. Beta-catenin binds to the activation function 2 region of the androgen receptor and modulates the effects of the N-terminal domain and TIF2 on ligand-dependent transcription. Mol Cell Biol. 2003;23(5):1674–87. Epub 2003/02/18. 12588987 10.1128/MCB.23.5.1674-1687.2003 PMC151689

[19] Mulholland DJ, Cheng H, Reid K, Rennie PS, Nelson CC. The androgen receptor can promote beta-catenin nuclear translocation independently of adenomatous polyposis coli. J Biol Chem. 2002;277(20):17933–43. Epub 2002/02/22. 10.1074/jbc.M200135200 M200135200 [pii]. . 11856748

[20] Yang F, Li X, Sharma M, Sasaki CY, Longo DL, Lim B, et al Linking beta-catenin to androgen-signaling pathway. J Biol Chem. 2002;277(13):11336–44. Epub 2002/01/17. 10.1074/jbc.M111962200 M111962200 [pii]. . 11792709

[21] Chesire DR, Isaacs WB. Ligand-dependent inhibition of beta-catenin/TCF signaling by androgen receptor. Oncogene. 2002;21(55):8453–69. Epub 2002/12/06 04:00. . 12466965 10.1038/sj.onc.1206049

[22] Cronauer MV, Schulz WA, Ackermann R, Burchardt M. Effects of WNT/beta-catenin pathway activation on signaling through T-cell factor and androgen receptor in prostate cancer cell lines. Int J Oncol. 2005;26(4):1033–40. Epub 2005/03/09 09:00. . 15753999 10.3892/ijo.26.4.1033

[23] Verras M, Sun Z. Roles and regulation of Wnt signaling and beta-catenin in prostate cancer. Cancer Lett. 2006;237(1):22–32. Epub 2005/07/19. S0304-3835(05)00549-5 [pii] 10.1016/j.canlet.2005.06.004 . 16023783

[24] Estebanez-Perpina E, Arnold LA, Nguyen P, Rodrigues ED, Mar E, Bateman R, et al A surface on the androgen receptor that allosterically regulates coactivator binding. Proc Natl Acad Sci U S A. 2007;104(41):16074–9. Epub 2007 Oct 2. 17911242 10.1073/pnas.0708036104 PMC1999396

[25] Yumoto F, Nguyen P, Sablin EP, Baxter JD, Webb P, Fletterick RJ. Structural basis of coactivation of liver receptor homolog-1 by beta-catenin. Proc Natl Acad Sci U S A. 2012;109(1):143–8. 10.1073/pnas.1117036108 22187462 PMC3252924

[26] Guex N, Peitsch MC, Schwede T. Automated comparative protein structure modeling with SWISS-MODEL and Swiss-PdbViewer: a historical perspective. Electrophoresis. 2009;30 Suppl 1(1):S162–73. Epub 2009/06/12 09:00. . 19517507 10.1002/elps.200900140

[27] Lin JF, Xu J, Tian HY, Gao X, Chen QX, Gu Q, et al Identification of candidate prostate cancer biomarkers in prostate needle biopsy specimens using proteomic analysis. Int J Cancer. 2007;121(12):2596–605. Epub 2007/08/28. 10.1002/ijc.23016 . 17722004

[28] Levy L, Wei Y, Labalette C, Wu Y, Renard CA, Buendia MA, et al Acetylation of beta-catenin by p300 regulates beta-catenin-Tcf4 interaction. Mol Cell Biol. 2004;24(8):3404–14. Epub 2004/04/03 05:00. . 15060161 10.1128/MCB.24.8.3404-3414.2004 PMC381622

[29] Mulholland DJ, Dedhar S, Coetzee GA, Nelson CC. Interaction of nuclear receptors with the Wnt/beta-catenin/Tcf signaling axis: Wnt you like to know? Endocr Rev. 2005;26(7):898–915. Epub 2005/08/30. er.2003-0034 [pii] 10.1210/er.2003-0034 . 16126938

[30] Storer CL, Dickey CA, Galigniana MD, Rein T, Cox MB. FKBP51 and FKBP52 in signaling and disease. Trends Endocrinol Metab. 2011;22(12):481–90. 10.1016/j.tem.2011.08.001 21889356 PMC3229651

[31] Jehle K, Cato L, Neeb A, Muhle-Goll C, Jung N, Smith EW, et al Coregulator control of androgen receptor action by a novel nuclear receptor-binding motif. J Biol Chem. 2014;289(13):8839–51. Epub 2014/02/14. M113.534859 [pii] 10.1074/jbc.M113.534859 24523409 PMC3979403

[32] Cluning C, Ward BK, Rea SL, Arulpragasam A, Fuller PJ, Ratajczak T. The helix 1–3 loop in the glucocorticoid receptor LBD is a regulatory element for FKBP cochaperones. Mol Endocrinol. 2013;27(7):1020–35. 10.1210/me.2012-1023 23686112 PMC5415241

[33] Cox MB, Riggs DL, Hessling M, Schumacher F, Buchner J, Smith DF. FK506-binding protein 52 phosphorylation: a potential mechanism for regulating steroid hormone receptor activity. Mol Endocrinol. 2007;21(12):2956–67. Epub 007 Aug 23. 17717070 10.1210/me.2006-0547

[34] Nair SC, Rimerman RA, Toran EJ, Chen S, Prapapanich V, Butts RN, et al Molecular cloning of human FKBP51 and comparisons of immunophilin interactions with Hsp90 and progesterone receptor. Mol Cell Biol. 1997;17(2):594–603. 9001212 10.1128/mcb.17.2.594 PMC231784

